# Transmission of Hereditary Diseases

**Published:** 1865-02

**Authors:** E. Nichols

**Affiliations:** Ann Arbor, Mich.


CHICAGO
MEDICAL JOURNAL.

VOL. XXII.] FEBRUARY, 1865. [No. 2.

### ORIGINAL COMMUNICATIONS.

### TRANSMISSION OF HEREDITARY DISEASES.

### ANIMAL GRAFTING—ORIGIN OF TUBERCULAR MATTER—SOLIDIST'S THEORY OF IT.

By E. NICHOLS, M. D.

Theories often arise and conclusions are drawn from very small and imperfect experiments and distant facts. Owing to this many arguments have been advanced without bestowing light on subjects discussed or being of practical benefit to any one. Among other questions discussed without obtaining satisfactory conclusions is that of the true *modus operandi* in the transmission of hereditary diseases; some holding that the transmission takes place through the solids, and others maintaining that the diseases are transmitted through the medium of the fluids.

For a long time I have had peculiar if not original ideas concerning the transmission of hereditary diseases, as well as the peculiarity of the commencement of certain maladies in the system. In the years 1861 and '62, I made some interesting experiments in *grafting animals*, and from them derived what I deemed a sufficient proof of my theories.

My idea was that the whole caste or peculiarity of a being belonging to a race or family rested with the *tissues* of the body—the *blood* or other fluids having nothing to do with it. I saw some crab apples growing on trees in the same soil that gave growth and nourishment to trees bearing sweet plumbs, while a tree loaded with rosy peaches stood in their midst. The soil furnished the same quality of nutriment to the roots of all these trees, yet their several saps varied in composition and nature. Then it must be a peculiarity in the roots of these trees—a difference in their selective powers—and not a difference in the nutriment furnished by the soil to each. By grafting, my theory was more closely proven. Place a very small piece of pear-sprig, a single bud, into the bark of a gnarled crabapple tree, and as fine a branch of pear and as sweet pears will grow on the crabapple-sap as could be grown on the parent tree. This, then, was proof positive that any peculiarity of a vegetable body rested with the *tissue* and *not* with the *fluid* that fed it. Was it the same with animal tissues? The question seemed interesting and well worth solving, and after a patient trial I succeeded in grafting animal tissue. I transplanted a small piece of integument from a squirrel into the skin of a dog, and it was retained and nourished after a time as well as if in its original place. *Squirrel skin and hair was grown from the blood of a dog.**

*In the year 1862 I began an article on hereditary diseases, in which I designed to mention this experiment; but, entering the army soon after, I never completed it. However, I wrote a short article of general interest and novelty in which I mentioned the result of my animal grafting, and which was published in a New York literary paper early A. D. 1862. Since that time Dr. Bert has published a book on "Animal Grafts," as I have been able to learn through the "Book Notices." I have not seen his work, nor do I know how far he claims originality, but I believe I am safe in saying that I am the first to publish any thing on this subject.

Nearly a year ago, I again commenced a course of experiments in animal grafting, and wrote to Prof. Ingals, of Rush Med. College, to enquire if he knew of ought to prevent my claiming originality in the experiments. His answer was, "that he knew of nothing ever having been published on the subject." Dr. Bert's book, I believe, has been issued since that date; yet Animal Grafting may be original with him as well.

Now, if peculiarities, whether predisposing to disease or superiority, was dependent on the fluids of the body, why did not the blood of a dog make dog-integument and dog-hair out of the squirrel skin depending on it for life and nourishment? According to the theories and belief of fluidists it should have done so. Because this did not occur, but on the contrary dog-blood built up squirrel tissue, I considered it a sufficient proof of this assersion. No matter what is the peculiarity of a certain tissue, all tissues arising out of it must partake of the same peculiarities, no matter what may be the characteristic nature of the fluid nourishing it. The assimulative power of the tissue will select only such material as will build up particles of the same nature, and its formative power will endow it with all its peculiar shapes and impressions. We know this by a more general view in observing that the same fluid feeds bone, muscle, ligaments, etc.

The germs for a new being we know are secreted from the blood of the parents; but in that germinal, or in the primative being there is no blood until it is generated by the tissues of the fœtus, while there is no interchange of blood with the mother and her offspring during the whole of fœtal life. Not only this, but a disease is as liable to be inherited from the father as the mother. Therefore, I can not understand how a disease may be transmitted by the blood. But if there is any peculiarity in the tissues of the parent, I can imagine how the germ may be marked by the same peculiarities, and the tissues of the new being endowed with the same distinguishing features. But when it comes to atoms too minute for the vision, even aided by the most powerful lens, it is difficult to determine their shape or nature, or understand the manner in which they are impressed. We can not observe molecular metamorphosis, yet it is these same minute molecules that bear the characteristic likenesses of its parents, and on the perfectness of these alone depends the tone and nature of the system they compose. If, owing to imperfection in the parents, they are in any way defective, they of course are liable, or predisposed to the same disease as were the parents. On the other hand, the blood does not depend on blood for its regeneration, and therefore can not for any length of time retain peculiarities in composition or hold in it diseased forms. Besides this there is no disease whatever existing in the system until there has been a deleterious change wrought in some of the molecules comprising it. Blood may be diseased; but the system does not suffer until the tissues themselves are changed or affected by it. The blood being imperfect may be the cause of changes in the tissues, and no doubt is often the cause of disease; yet perhaps the blood is more dependent for its qualities on some of the tissues than we universally maintain. Blood, being a medium of elimination, I can not believe it capable of retaining a disease, though at times it may have in it bodies foreign to its nature. If these are of a nature not allowing of elimination through the excretory glands, the blood will not long retain them, but deposit them in some organ where they may then produce disease.

I have maintained that when a particle of tissue is disintegrated and replaced, that the new particles bear the same distinguishing features as marked its predecessor; yet I would not be understood to believe that the impressions do not grow less and less marked as molecular changes go on in a healthy and tonic condition. Yet that these impressions are retained by the tissues, there is little room for doubt. How else can *memory by the brain* be accounted for? Particles of the brain receive impressions from the external world through the special senses.

The particles impressed, however, are constantly going through the same metamorphosis as other molecules, and unless the successive particle was built up with the same peculiar impression the *memory* would be destroyed with the atom of brain matter disintegrated.

Taking this view of the subject, I will glance at the commencement of an acknowledged hereditary disease in a system and trace out the *origin of Tubercular matter*.

Tubercular matter, such as is deposited in the lungs in phthisis tuberculosis, differs from any of the tissues of the body, and it is difficult to decide accurately regarding its origin, and our theories must of necessity be more or less hypothetical; but often hypothetical arguments work out well in practice.

Every action of the body is the cause of decay to the tissues, and this constant disintegration is counteracted by a process of perpetual rebuilding. The disintegrated matter is eliminated by exosmosis from the cell-space into the capillaries, and a proper supply of new building material is taken up by an assimulative power of the changing molecule. If nature has a free scope of operation the matter endosmosed from the capillaries to the cell-space will correspond in quantity and quality to the matter broken down and exosmosed. But there may be an interruption of this natural process; and then there may be other matter taken up from the blood than such as can be converted into tissue of that kind, or the disintegrated matter may fail to pass from the cell-space into the capillaries, owing to an arrest of circulation in the capillaries, or otherwise.

Consumption is liable to a system where disintegration occurs more rapidly than the process of rebuilding can take place, whether the causes are hereditary or not. There are many causes which produce it in systems not predisposed to it; but I will confine my brief notes to the hereditary form only.

The *peculiarity* of a tissue-molecule predisposing to the formation of tuburcular matter I will suppose to be a lacking in its assimulative power; or, being imperfectly formed, it may be more rapidly torn down than a healthy molecule, and therefore has less power to select proper nutriment for its reproduction. This may give rise to a foreign matter being placed in the cell that should be occupied by a perfect tissue molecule. This foreign matter may be the disintegrated part of the old molecule which has not been thrown out, owing to an interruption of the laws of exosmosis and endosmosis, or it may be a conglomerated material from the blood, which has passed into the cell through other agencies than by the assimulative power. This matter is surrounded by chemical changes which will cause it to endeavor to take on an organic nature, and as it is not proper material for tissues, it becomes a semi-organic body—*tubercle.* This occupies the cell-space and no doubt takes up matter for its growth like all other molecules, and as it increases in size it is forced from its lodgement if situated in an active organ—muscle for instance—and is carried off in the blood. If not too large it is eliminated through an excretory organ; but, if too large, it is carried on until it finds lodgement in the capillaries of an organ that is not active and hardy enough to displace it and force it on in the circulation. If it stops in the liver, or lungs, or like structures, it remains, and by its assimulative power for like material it increases in size until it makes a large tubercle. The reason we have this matter deposited in the lungs more frequently than elsewhere is no doubt because all the blood of the circulation passes through these organs, and because there is less force here to expel the foreign matter.

Thus we have the formation of tubercles owing to a predisposition of the *tissues* to a *too rapid disintegration*, or a *lack of assimulative power*. Yet, can there not be an improvement wrought in such tissues and the results of such conditions warded off? Without doubt, I think, there can be improvement made and the tissues restored to a healthy condition. Let us again have recourse to the mind and brain for illustration. It may appear to be getting into a shadowy realm for illustration; but I hope the investigation will be none the less satisfactory.

We have said that a particle of brain matter being impressed, its successor is impressed in like manner. This must be so, as a thing once received by the brain may not be acted upon for a long time by *nerve-fluid*, or whatever else it may be—known as such though,—yet the impression is still there, though perhaps less perfect than when first received. And, owing to this very imperfectness, we must believe that each succeeding particle is less strongly impressed until lost entirely, and we have instead of memory a thing forgotten. Thus, for instance, you will see in two old friends who have not met for many years, that one can recount incidents in the early lives of both that the other can not recall a shadow of, and *vice versa*. This is simply because one has not thought of it since its occurrence, while the other has had it frequently reimpressed by the action of thought. So it is with tissue molecules. Though deleteriously impressed at first, if all the agencies which caused this impression are studiously avoided so that no reimpression will occur—which will take place much easier than in the first instance—the molecules may be brought back to a normal condition.

Knowing the causes of phthisis tuberculosis, it will not, then, be difficult to prescribe hygiene for one predisposed to it, and in like manner we draw our inferences in regard to the treatment of those already suffering from this dreadful malady.

Ann Arbor, Mich., Jan. 7th, 1865.

